
JOURNAL INFORMATION
==============================
NLM Title Abbreviation: Wirtschaftsdienst
Journal ID: Wirtschaftsdienst
NLM Journal ID: 101086612

Wirtschaftsdienst (Hamburg, Germany : 1949)

ISSN: 0043-6275

ARTICLE INFORMATION
==============================
PMCID: PMC7765695
PMID: 33390625
DOI: 10.1007/s10273-020-2807-z
Article ID: 2807
Article version: 1
Subjects: Ökonomische Trends

Großelternbetreuung und COVID-19

Grandparent care and COVID-19

Nikolka Till nikolka@dji.de

Boll Christina boll@dji.de

grid.424214.5 0000 0001 1302 5619 Abteilung Familie und Familienpolitik, Deutsches Jugendinstitut e.V., Nockherstraße 2, 81541 München, Deutschland
Electronic publication date: 2020 Dec 26
Print publication date: 2020
Volume: 100
Issue: 12
First page: 976
Last page: 978
Copyright: © Der/die Autor(en) 2020
License: Dieser Artikel wird unter der Creative Commons Namensnennung 4.0 International Lizenz (https://creativecommons.org/licenses/by/4.0/deed.de) veröffentlicht.
Open Access wird durch die ZBW — Leibniz-Informationszentrum Wirtschaft gefördert.
License URL: https://creativecommons.org/licenses/by/4.0/

issue-copyright-statement: © The Author(s) 2020

==============================
Dr. Christina Boll leitet am Deutschen Jugendinstitut in München die Abteilung Familie und Familienpolitik und ist Gastprofessorin für Volkswirtschaftslehre an der Hochschule der Bundesagentur für Arbeit (HdBA).

Seit Beginn der COVID-19-Pandemie steht die Frage im Raum, inwieweit Generationenbeziehungen und insbesondere innerfamiliale Kontakte zwischen Großeltern und Enkeln die Infektionsrisiken der älteren Bevölkerung erhöhen könnten. Die Rolle innerfamilialer Kontakte für das Infektionsgeschehen wurde inzwischen in einer Reihe von Studien untersucht (Aparicio und Grossbard, 2020; Arpino et al., 2020; Balbo et al., 2020; Bayer und Kuhn, 2020). Aufgrund der höheren Mortalitätsrate Älterer haben bereits frühe Studien auf regional unterschiedlich verteilte Risiken, z.B. bedingt durch die unterschiedliche Alterskomposition der Bevölkerung (Kashnitsky und Aburto, 2020) oder unterschiedlich ausgeprägte Generationenbeziehungen (Balbo et al., 2020) hingewiesen. Studien für die USA stellen einen positiven Zusammenhang zwischen der Häufigkeit generationenübergreifender Koresidenz und coronabezogenen Todesfällen pro Kopf her (Aparicio und Grossbard, 2020). Zu ähnlichen Ergebnissen kommen Bayer und Kuhn (2020) auf Basis einer ländervergleichenden Studie für 24 Länder. Arpino et al. (2020) finden jedoch in ihren Analysen von Generationenbeziehungen in verschiedenen europäischen Ländern auf Basis von SHARE-Daten keinen Beleg für einen solchen Zusammenhang.

Analysen auf Basis deutscher Daten, die in tieferer regionaler Gliederung als bei vorangehenden Studien der Fall einen Zusammenhang zwischen Großelternbetreuung und Infektionszahlen motivieren könnten, liegen bisher nicht vor. Wir schließen diese Lücke, indem wir den Zusammenhang zwischen Großelternbetreuung und der Infektionsrate pro 100.000 Einwohner in der Altersgruppe 60+ auf Kreisebene untersuchen. Basierend auf Altersgruppenvergleichen zur SARS-CoV-2-Mortalitätsrate zeigen Kashnitsky und Aburto (2020), dass Personen im Alter 60+ besonders vulnerabel sind. Wir verwenden SARS-CoV-2-Infektionsraten der deutschen Gesundheitsämter für diese Altersgruppe auf Kreisebene (RKI, 2020). Die Infektionsraten in unserer Analyse beziehen sich auf alle registrierten Infektionen bis zum 23. März 2020. Dies war der Tag, als die Kontaktbeschränkungen in Deutschland in Kraft traten. Unsere Ergebnisse sind jedoch robust, wenn stattdessen die Infektionszahlen bis 30. September 2020 verwendet werden. Für den Zusammenhang zur Großelternbetreuung verwenden wir Mikrodaten der 6. Welle der Kinderbetreuungsstudie (KiBS) des Deutschen Jugendinstituts (Alt et al., 2020; Aust et al., 2018). Die Befragungsdaten wurden 2017 erhoben und umfassen 36.800 Auskunftspersonen zu Kindern im Alter von 0 bis 14 Jahren in 249 deutschen Kreisen. Der Wortlaut der Frage in der KiBS-Befragung ist: „In welchem Umfang wird Ihr Kind von den Großeltern betreut?“ Die Antwortmöglichkeiten sind: „regelmäßig“, „nach Bedarf“ und „gar nicht“. Für unsere Analysen nutzen wir die Zustimmung zur erstgenannten Kategorie „regelmäßig“. Als dritte Datenquelle nutzen wir administrative Daten auf Kreisebene, des interaktiven Online-Atlas INKAR (Indikatoren und Karten zur Raum- und Stadtentwicklung; BBSR, 2020).

Da Großelternbetreuung nur eine Kontaktquelle unter vielen ist, beziehen sich unsere untersuchungsleitenden Hypothesen auf die regelmäßige Großelternbetreuung und deren möglichen Bezug zu den Infektionsraten Älterer auf Kreisebene sowie auf eine Reihe konfundierender Faktoren dieses Zusammenhangs. Unser Untersuchungsansatz unterteilt sich demnach in zwei Bereiche, erstens die Erklärung der Nutzung von regelmäßiger Großelternbetreuung auf der Mikroebene und zweitens die Erklärung der Infektionsraten Älterer auf Kreisebene unter Einbindung der auf Kreisebene aggregierten Nutzung von regelmäßiger Großelternbetreuung. Untersuchungsstrang 1 sind die Hypothesen H1 und H2, Untersuchungsstrang 2 die Hypothesen H3 bis H9 zugeordnet.

Bevölkerungsanteil der Katholik*innen auf Kreisebene ein Indikator für einen starken innerfamilialen Zusammenhalt ist, der die Inanspruchnahme von Großelternbetreuung auf der Mikroebene der Familie wahrscheinlicher macht (H2).

Für die Infektionsrate in der Bevölkerung im Alter 60+ auf Kreisebene nehmen wir an, dass diese erstens mit dem Infektionspfad, den wir seit dem ersten registrierten Corona-Infektionsfall auf Kreisebene messen (H3), zweitens mit der Siedlungsdichte, die wir mit vier siedlungsstrukturellen Kreistypen erfassen (H4), und drittens mit der individuellen Kontaktfrequenz steigen sollte. Letztere sollte wiederum von mehreren Faktoren getrieben sein. So sollte die Infektionsrate ceteris paribus mit mehr Kontakten steigen, im Zusammenhang mit der Inanspruchnahme institutioneller Kinderbetreuung (gemessen in kreisspezifischen Betreuungsquoten U3 bzw. drei bis fünf Jahre; H5), einer höheren wirtschaftlichen Aktivität, die wir mit dem Medianeinkommen auf Kreisebene operationalisieren (H6), und einem höheren Anteil der Kinder unter 15 Jahren auf Kreisebene, der regelmäßig von Großeltern betreut wird (H7). Bisherige Studien deuten darauf hin, dass infizierte Kinder ohne eigene Krankheitssymptome zur Verbreitung des Virus beitragen könnten (Hippich et al., 2020; Laxminarayan et al., 2020).

Neben dem Mechanismus stärkerer innerfamilialer Verbindungen könnte eine katholische Religionszugehörigkeit auch über einen außerfamilialen Kanal sozialer Netzwerke die Infektionsrate treiben (H8), z. B. über Feierlichkeiten mit religiösem Bezug wie Gottesdienste oder Hochzeiten (Lee et al., 2020; Salvador et al., 2020). Im Februar 2020 könnten auch Karnevalsaktivitäten in Rheinland-Pfalz und Nordrhein-Westfalen im Zusammenhang mit dem Infektionsgeschehen gestanden haben. Zudem nehmen wir an, dass ein geringerer Bevölkerungsanteil Älterer (60+) und ein höherer Anteil von Personen unter 18 Jahren mit einer höheren Infektionsrate zusammenhängen könnte (H9). Als weitere Kontrollvariablen fügen wir den Ausländeranteil auf Kreisebene sowie einen Dummy für ostdeutsche Kreise in das empirische Modell ein.

Wie deskriptive Analysen zeigen, variierten die Infektionszahlen pro 100.000 Einwohner im Alter 60+ am 23. März 2020 zwischen 24,2 (Mecklenburg-Vorpommern) und 185,6 (Baden-Württemberg). Regelmäßige Großelternbetreuung ist in den nördlichen Bundesländern sowie in den Stadtstaaten weniger verbreitet als in den südlichen Bundesländern. Der Anteil regelmäßiger Betreuung schwankt im Bundesgebiet zwischen 11,9 % (Mecklenburg-Vorpommern) und 18,3 % (Thüringen) und ist in kreisfreien Großstädten (13,2 %) niedriger als in ländlichen Kreisen (18,5 %).

Als erste multivariate Analyse schätzen wir die Wahrscheinlichkeit regulärer Großelternbetreuung als lineares Wahrscheinlichkeitsmodell auf einer gewichteten Stichprobe von 33.259 Kindern im Alter von 0 bis 14 Jahren, mit folgenden Ergebnissen: Verglichen mit einem Paarhaushalt mit männlichem Alleinverdiener, ist reguläre Großelternbetreuung bei alleinerziehenden Müttern ungeachtet ihres Erwerbsstatus, bei alleinerziehenden erwerbstätigen Vätern sowie bei Paarhaushalten, in denen beide Eltern oder nur die Mutter erwerbstätig sind/ist, stärker verbreitet. Ebenfalls wird Großelternbetreuung erwartungsgemäß bei Inanspruchnahme institutioneller Kinderbetreuung seltener und bei einem jüngsten Kind im Alter von drei bis fünf Jahren häufiger als in allen anderen Altersgruppen genutzt. Die Ergebnisse zum Haushaltskontext (H1) bestätigen damit weitgehend unsere Erwartungen. Zudem geht, H2 bestätigend, ein 10 %iger Anstieg des Katholikenanteils auf Kreisebene mit einem signifikanten Anstieg der regulären Großelternbetreuung um 0,4 bis 0,6 Prozentpunkte einher.

Zweitens schätzen wir die kreisspezifische Infektionsrate in der Bevölkerung 60+ pro 100.000 Einwohner ebenfalls als lineares Regressionsmodell. Wir schätzen unterschiedliche Varianten dieses Modells, wobei wir neben dem Infektionspfad (Tage seit erstem registrierten Fall) und dem Anteil regelmäßiger Großelternbetreuung als unsere Kernvariable weitere Faktoren sukzessive einführen. Es zeigt sich, dass der Infektionspfad in allen Spezifikationen signifikant positiv mit der Infektionsrate verbunden ist: Je früher der erste registrierte Fall in einem Kreis auftrat, je höher die Infektionsrate am 23. März 2020, was Hypothese H3 bestätigt. Kreise mit höherer ökonomischer Aktivität sowie städtische Kreise gehen mit höheren Infektionsraten in der älteren Bevölkerung einher. Dies stützt unsere Hypothesen H4 und H6. Hingegen sind die Betreuungsquoten in der Kinderbetreuung und der Anteil Älterer (60+) nicht signifikant und der Bevölkerungsanteil der unter 18-Jährigen nicht durchgängig mit der Infektionsrate des Kreises assoziiert, weshalb unsere Hypothesen H5 und H9 teilweise verworfen werden müssen.

Der Anteil regelmäßiger Großelternbetreuung ist unter Einbindung aller bisher genannten Variablen signifikant positiv mit der Infektionsrate verbunden, verliert aber bei Addition des Bevölkerungsanteils der Katholiken seine Signifikanz, sodass H7 zunächst scheinbar bestätigt wird, im vollständigen Modell aber nicht. Der Katholikenanteil ist hochsignifikant positiv, was Hypothese H8 bestätigt. Der Parameter der Großelternbetreuung wird in allen getesteten Spezifikationen insignifikant, sobald wir für bundeslandfeste Effekte kontrollieren. Dies deutet an, dass der Zusammenhang zwischen Großelternbetreuung und Infektionsraten auf Kreisebene vor allem durch Unterschiede zwischen den Bundesländern getrieben wird. Der Katholikeneffekt über die außerfamilialen Netzwerke sollte eine höhere Relevanz haben, wenn eine ausreichende Zahl

Abbildung 1
**Zusammenhang von COVID-19-Infektionen Älterer mit der Betreuung von Enkelkindern**

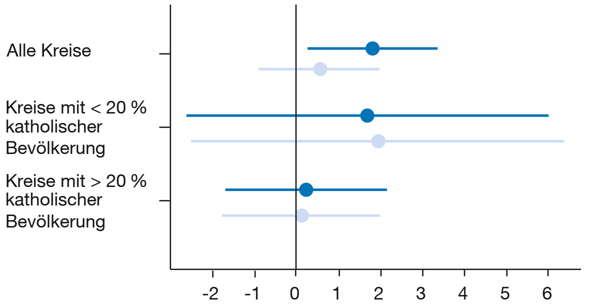

Anmerkungen: Abhängige Variable: Log Infektionen pro 100.000 Ew. 60+ (bis 23. März 2020); Koeffizient: Anteil regelmäßige Betreuung (U15) durch Großeltern. Weitere Regressoren: Log(Tage seit Patient 1), Log(Bev./km2), Log(Medianeinkommen), Dummy: Ostdeutschland, Dummy: Städtische Kreise, Anteil Ew. U60, Anteil Ew. U18, Betreuungsquote U3, Betreuungsquote 3-5, Ausländeranteil. Gezeigt wird der Koeffizient und das 95 % Konfidenzintervall für den Anteil der regelmäßigen Großelternbetreuung (U15), bevölkerungsgewichtete OLS-Schätzung auf Kreisebene in Westdeutschland.

Quelle: Eigene Berechnungen auf Basis von KiBS Surveydaten des Deutschen Jugendinstituts sowie amtlichen Daten des BBSR (2020) und RKI (2020).

von Peers im Kreis vorhanden ist. Wir testen diese Hypothese durch eine Splittung unserer Stichprobe in Kreise mit einem Katholikenanteil von über und unter 20 %. Da alle ostdeutschen Kreise in die letztgenannte Gruppe gehören, beschränken wir die Analyse auf westdeutsche Kreise. Für die Gruppe der Kreise mit weniger als (mindestens) 20 % Katholikenanteil nehmen wir an, dass der Mechanismus der außerfamilialen Netzwerke hier vergleichsweise schwächer (stärker) und der innerfamiliale Mechanismus vergleichsweise stärker (schwächer) ausfällt.

Die Ergebnisse zeigen, dass Großelternbetreuung in keiner der beiden Gruppen signifikant mit den Infektionsraten auf Kreisebene zusammenhängt. Auch im Südwesten Deutschlands also, wo die meisten Katholiken wohnen, steht die reguläre Großelternbetreuung nicht signifikant mit den Infektionen in Zusammenhang. Allerdings zeigen die dunkelblauen Punkte der Koeffizienten-Plots in Abbildung 1, dass die Effektgröße der Großelternbetreuung erwartungsgemäß in Kreisen mit unter 20 % Katholikenanteil höher ist als in der anderen Kreisgruppe. Wird in diesen Substichproben zusätzlich der Katholikenanteil kontrolliert (hellblaue Punkte), steigt der Koeffizient der Großelternbetreuung in Kreisen mit wenig Katholiken leicht an, bleibt aber insignifikant. In der anderen Kreisgruppe nimmt er weiter ab. Der obere blaue bzw. hellblaue Punkt bildet den Koeffizienten der Großelternbetreuung basierend auf der gesamten Kreisstichprobe ab. Unsere Daten bestätigen die These nicht, dass es einen validen eigenständigen Zusammenhang zwischen Großelternbetreuung und Infektionszahlen auf Kreisebene gibt. Obwohl der Katholikenanteil auf Kreisebene die Inanspruchnahme von Großelternbetreuung begünstigt, zeigen die Regressionen der kreisspezifischen Infektionsrate, dass der Effekt des Katholikenanteils bestehen bleibt, auch wenn der Effekt der Großelternbetreuung kontrolliert wird; während der Effekt der Großelternbetreuung dann absorbiert wird, bleibt der Katholikeneffekt signifikant. Wir vermuten, dass hierfür soziale Netzwerke außerhalb der Familie verantwortlich sind, die allerdings nicht gemessen wurden, sodass auch andere regionsspezifische Effekte eine Rolle spielen könnten. Der testweise Einbezug von bundeslandfesten Effekten deutet darauf hin, dass die Variation eher zwischen Bundesländern als zwischen Kreisen desselben Bundeslandes verläuft. In jedem Fall steht fest: Die Hypothese, nach der die Großelternbetreuung eine Mitschuld am Infektionsgeschehen trifft, lässt sich anhand von Regionaldaten nicht bestätigen.


Dr. Till Nikolka ist dort wissenschaftlicher Referent.


Literatur

Alt, C., J. Anton, B. Gedon, S. Hubert, K. Hüsken, K. Lippert und V. Schickle (2020), DJI-Kinderbetreuungsreport 2019. Inanspruchnahme und Bedarf aus Elternperspektive im Bundesländervergleich, DJI.
Althammer, J. (Hrsg.) (2013), Caritas in veritate. Katholische Soziallehre im Zeitalter der Globalisierung, Duncker & Humblot.
Aparicio, A. und S. Grossbard (2020), Intergenerational residence patterns and COVID-19 fatalities in the EU and the US, ZA Discussion Paper, 13452.10.1016/j.ehb.2020.100934 PMC7598770 33160264
Arpino, B., V. Bordone und M. Pasqualini (2020), No clear association emerges between intergenerational relationships and COVID-19 fatality rates from macro-level analyses, Forthcoming in Proceedings of the National Academy of Sciences (PNAS). 10.1073/pnas.2008581117 PMC7431085 32699150
Aust, F., J. v. d. Burg, D. Hess und B. Knecht (2018), Methodenbericht — Kinderbetreuungsstudie 2017 und Zusatzuntersuchung, Pilotstudie: Räumliche Mobilität von Familien und Kindern in Zeiten der Digitalisierung.
Balbo, N., F. C. Billari und A. Melgrano (2020), The strength of family ties and COVID-19, Contexts, American Sociological Association, https://contexts.org/blog/structural-shocks-and-extreme-exposures/#balbo (3. Dezember 2020).
Bayer, C. und M. Kuhn (2020), Intergenerational ties and case fatality rates: a cross-country analysis, ZA Discussion Paper, 13114.
BBSR (Bundesinstitut für Bau-, Stadt- und Raumforschung) (2020), Indikatoren und Karten zur Raum- und Stadtentwicklung (INKAR).
Gundlach, G. (1964), Die Ordnung der menschlichen Gesellschaft, 2 Bde.
Hippich M Holthaus L Assfalg R Zapardiel Gonzalo J M Kapfelsperger H Heigermoser M Haupt F Ewald D A Welzhofer T C Marcus B A Heck S Koelln A Stock J Voss F Secchi M Piemonti L Rosa K Protzer U Boehmer M Achenbach P Lampasona V Bonifacio E Ziegler A G Public health antibody screening indicates a six-fold higher SARS-CoV-2 exposure rate than reported cases in children Med 2020 2 1 15 10.1016/j.medj.2020.10.003 PMC7598360 33163984
Kashnitsky, I. und J. M. Aburto, (2020), COVID-19 in unequally ageing European regions, World Development. 10.1016/j.worlddev.2020.105170 PMC7455200 32895594
Laxminarayan R Wahl B Dudala S R Gopal K Neelima S Reddy K J Radhakrishman J Lewnard J A Epidemiology and transmission dynamics of COVID-19 in two Indian states Science 2020 370 6517 691 697 10.1126/science.abd7672 33154136 PMC7857399
Lee E C Wada N I Grabowski M K Gurley E S Lessler J The engines of SARS-CoV-2 spread Science 2020 370 6515 406 407 10.1126/science.abd8755 33093098
RKI (Robert Koch-Institut) (2020), Fallzahlen in Deutschland, dl-de/by-2-0.
Salvador C Berg M Yu Q Alvaro S M Kitayama S Relational Mobility Predicts Faster Spread of COVID-19: A 39-Country Study Psychological Science 2020 31 10 1236 1244 10.1177/0956797620958118 32915703
